# Onset of herbivore-induced resistance in systemic tissue primed for jasmonate-dependent defenses is activated by abscisic acid

**DOI:** 10.3389/fpls.2013.00539

**Published:** 2013-12-30

**Authors:** Irene A. Vos, Adriaan Verhage, Robert C. Schuurink, Lewis G. Watt, Corné M. J. Pieterse, Saskia C. M. Van Wees

**Affiliations:** ^1^Plant-Microbe Interactions, Department of Biology, Faculty of Science, Utrecht UniversityUtrecht, Netherlands; ^2^Department of Plant Physiology, Swammerdam Institute for Life Sciences, University of AmsterdamAmsterdam, Netherlands

**Keywords:** abscisic acid, jasmonic acid, priming, systemic defense, *Pieris rapae*, MYC2

## Abstract

In *Arabidopsis*, the MYC2 transcription factor on the one hand and the AP2/ERF transcription factors ORA59 and ERF1 on the other hand regulate distinct branches of the jasmonic acid (JA) signaling pathway in an antagonistic fashion, co-regulated by abscisic acid (ABA) and ethylene, respectively. Feeding by larvae of the specialist herbivorous insect *Pieris rapae* (small cabbage white butterfly) results in activation of the MYC-branch and concomitant suppression of the ERF-branch in insect-damaged leaves. Here we investigated differential JA signaling activation in undamaged systemic leaves of *P. rapae*-infested plants. We found that the *MYC2* transcription factor gene was induced both in the local insect-damaged leaves and the systemic undamaged leaves of *P. rapae*-infested *Arabidopsis* plants. However, in contrast to the insect-damaged leaves, the undamaged tissue did not show activation of the MYC-branch marker gene *VSP1*. Comparison of the hormone signal signature revealed that the levels of JA and (+)-7-*iso*-jasmonoyl-L-isoleucine raised to similar extents in locally damaged and systemically undamaged leaves, but the production of ABA and the JA precursor 12-oxo-phytodienoic acid was enhanced only in the local herbivore-damaged leaves, and not in the distal undamaged leaves. Challenge of undamaged leaves of pre-infested plants with either *P. rapae* larvae or exogenously applied ABA led to potentiated expression levels of *MYC2* and *VSP1*, with the latter reaching extremely high expression levels. Moreover, *P. rapae*-induced resistance, as measured by reduction of caterpillar growth on pre-infested plants, was blocked in the ABA biosynthesis mutant *aba2-1*, that was also impaired in *P. rapae*-induced expression of *VSP1*. Together, these results suggest that ABA is a crucial regulator of herbivore-induced resistance by activating primed JA-regulated defense responses upon secondary herbivore attack in *Arabidopsis*.

## INTRODUCTION

Plants possess sophisticated defense mechanisms to protect themselves against pathogens and herbivorous insects. These mechanisms include structural and chemical barriers, that can be constitutively present in the plant or can be induced upon activation of the plant immune system. Recognition of the attacking organism induces local defense responses and the resistance induced is often extended to systemic tissue, thereby protecting undamaged distal plant parts against future attack. The phytohormones jasmonic acid (JA) and salicylic acid (SA) are major regulators of the induced defense signaling network controlling local as well as systemic resistance signaling events in roots and leaves (Pieterse et al., 2012; Soler et al., 2013). The SA–JA backbone of the immune signaling network can be modified by other hormones, such as ethylene (ET) and abscisic acid (ABA; Van Loon et al., 2006; Ton et al., 2009; Robert-Seilaniantz et al., 2011). The hormone signal signature produced upon pathogen or insect attack depends on the stimuli perceived and determines the suite of attacker-specific defense responses that are activated in the plant (De Vos et al., 2005; Pieterse et al., 2009; Verhage et al., 2010).

Jasmonic acid is an important hormone regulating the induction of defense responses to herbivorous insects and necrotrophic pathogens (Glazebrook, 2005; Howe and Jander, 2008). Infestation of *Arabidopsis* with larvae of the specialist chewing herbivore *Pieris rapae* (small cabbage white butterfly) induces defense responses that inhibit *P. rapae* performance, resulting in reduced weight gain of the caterpillars on pre-infested plants (De Vos et al., 2006). In many plant species it has been shown that this wound-induced resistance also extends systemically to undamaged plant parts (Howe and Jander, 2008). JA or one of its isoforms have been implicated as important signals in both root- and shoot-induced systemic defenses upon herbivory in various plant–herbivore interactions (Green and Ryan, 1972; Howe and Jander, 2008; Soler et al., 2013). Depending on the hormonal context and the below- or above-ground origin of JA, different JA-dependent responses in systemic tissues are activated (Pieterse et al., 2012; Tytgat et al., 2013).

Disruption of plant tissue by herbivory triggers production of JA and its structurally related oxylipin derivatives (collectively called jasmonates (JAs); Mithöfer et al., 2005). The F-box protein COI1 (CORONATINE INSENSITIVE1) functions as a key regulator of JA signaling (Xie et al., 1998). Mutant *coi1-1* plants are unresponsive to JAs and show alterations in the level of resistance to different herbivorous insects and necrotrophic pathogens (Van der Ent et al., 2009b). (+)-7-*iso*-jasmonoyl-L-isoleucine (JA-Ile) has been determined as the most biologically active form of JA (reviewed in Wasternack and Hause, 2013), however, the role of other oxylipin isoforms in activation of JA signaling has remained largely unknown. Work on OPR3-impaired *opr3* mutants revealed that the JA-precursor 12-oxo-phytodienoic acid (OPDA) is a direct regulator of a distinct set of JA-responsive genes (Stintzi and Browse, 2000; Taki et al., 2005; Böttcher and Pollmann, 2009), suggesting that additional oxylipins influence the final outcome of the JA response. Within the JA signaling pathway, two distinct, antagonistic branches of transcriptional regulation by JA are recognized. The ETHYLENE RESPONSE FACTOR (ERF)-branch, which is co-regulated by ET, is activated upon infection with necrotrophic pathogens and is controlled by the AP2/ERF domain transcription factors ERF1 and ORA59, leading to transcription of *PDF1.2*, a marker gene of the JA/ET-regulated ERF-branch (Penninckx et al., 1998; Lorenzo et al., 2004; Pré et al., 2008). The MYC-branch, which is co-regulated by ABA, is activated upon feeding by herbivorous insects and is regulated by the basic helix-loop-helix leucine zipper proteins MYC2, MYC3, and MYC4, leading to the transcription of *VSP1* and *VSP2* marker genes (Anderson et al., 2004; Thaler and Bostock, 2004; Lorenzo and Solano, 2005; Fernández-Calvo et al., 2011; Niu et al., 2011).

ABA is known to have synergistic effects on the MYC-branch and antagonistic effects on the ERF-branch, as evidenced by effects of ABA on JA-induced transcriptional activation which was enhanced by ABA for *MYC2* and *VSP2* but suppressed by ABA for *ERF1*, *ORA59*, and *PDF1.2* (Anderson et al., 2004; Kazan and Manners, 2013). In line with these findings, ABA-deficient mutants were reported to be more susceptible to herbivory (Thaler and Bostock, 2004; Bodenhausen and Reymond, 2007) and more resistant to necrotrophic pathogens (Anderson et al., 2004; Sánchez-Vallet et al., 2012). In *Nicotiana attenuata* plants, ABA has been shown to amplify JA-dependent defense responses as part of the signal transduction pathway that is elicited by oral secretions of *Manduca sexta* larvae (Dinh et al., 2013). There have also been several reports on the role of ABA in systemic induced resistance triggered by diverse stimuli. It was hypothesized that ABA may function as a systemic signal in mediating above-ground resistance triggered by below-ground herbivory in maize (Erb et al., 2009). However, it was proven in a subsequent study that the enhanced level of resistance was independent of ABA and instead was due to induced water stress in the plant upon root herbivory (Erb et al., 2011). ABA was demonstrated to have a role in induced systemic resistance (ISR) that is elicited by below-ground beneficial rhizobacteria in *Arabidopsis* (Van der Ent et al., 2009a). ABA signaling was involved in priming of above-ground defenses, as evidenced by enhanced callose deposition upon challenge of ISR-expressing tissue with the pathogen *Hyaloperonospora arabidopsidis*. A similar role for ABA was shown for induced resistance triggered by β-aminobutyric acid (BABA; Ton et al., 2005).

In *Arabidopsis*, feeding by *P. rapae* larvae results in activation of the MYC-branch and concomitant suppression of the ERF-branch of the JA pathway in insect-damaged leaves (Verhage et al., 2011). In two-choice assays with *P. rapae* larvae and *Arabidopsis* plants it was shown that the caterpillars preferred to feed from plants that express the ERF-branch of the JA pathway over plants that express the MYC-branch (Verhage et al., 2011). This suggests that suppression of the ERF-branch by activating the MYC-branch is part of the plant’s defense strategy in this interaction. Here, we investigated the engagement of the MYC- and ERF-branches in *P. rapae*-induced resistance in undamaged leaves. We provide evidence that undamaged leaves of herbivore-infested plants express elevated levels of *MYC2* mRNA, resulting in priming of the MYC-branch of the JA pathway. The enhancement in ABA levels, like upon secondary herbivore attack, in the primed leaves mediates a potentiated expression of the MYC-branch resulting in enhanced expression levels of *VSP1*. This is associated with enhanced herbivore resistance in previously infested plants, which is shown to be ABA-dependent.

## RESULTS

### EFFECTS OF *P. rapae* FEEDING ON DIFFERENTIAL JA-REGULATED RESPONSES IN DISTAL TISSUE

**FIGURE 1:**
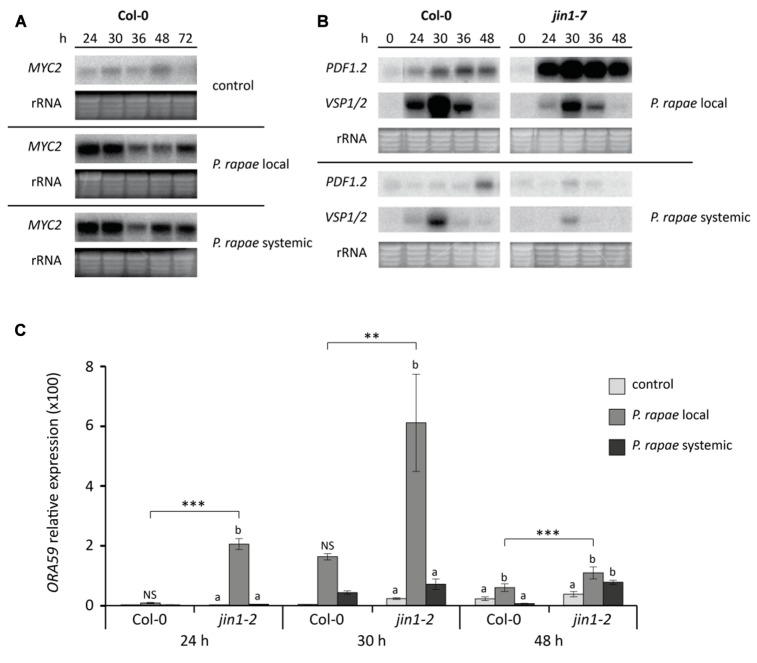
**Differential JA responses in Col-0 and MYC2-impaired *jin1* plants in local damaged and systemic undamaged leaves upon herbivory by *P. rapae*. (A)** Northern blot analysis of *MYC2* transcript levels in non-infested (control) and *P. rapae*-infested Col-0 plants. First-instar larvae were allowed to feed for 24 h after which they were removed (*t* = 24 h). Subsequently, damaged (*P. rapae* local) and undamaged (*P. rapae* systemic) leaf tissue was harvested for gene expression analysis. **(B)** Northern blot analysis of JA-responsive *PDF1.2* (marker for ERF-branch) and *VSP1/2* (marker for MYC-branch) gene expression in damaged (*P. rapae* local) and undamaged (*P. rapae* systemic) tissue of *P. rapae*-infested Col-0 and MYC2-impaired *jin1-7* plants. **(C)** RT-qPCR analysis of *ORA59* transcript levels (relative to non-infested Col-0 at 24 h) in non-infested (control) and *P. rapae*-damaged (*P. rapae* local) and -undamaged (*P. rapae* systemic) tissue of infested Col-0 and *jin1-2* plants. Asterisks indicate statistically significant differences between genotypes at specific time points (**P < 0.01; ***P < 0.001) and different letters indicate statistically significant differences between treatments within one genotype at specific time points (*P* < 0.05; NS, not significant). Data were analyzed per time point using two-way ANOVA.

In *Arabidopsis*, *P. rapae* feeding locally activates the MYC-branch of the JA pathway while the ERF-branch is suppressed (Verhage et al., 2011). To investigate the expression of this differential JA response in undamaged (systemic) leaves of *P. rapae*-infested plants, we monitored the expression of the key transcription factor genes *MYC2* and *ORA59*, as well as their respective marker genes *VSP1/2* and *PDF1.2*. The probe used for detection of *VSP* gene expression on northern blots was found to detect both *VSP1* and *VSP2* and the expression is therefore designated *VSP1/2*. Based on RT-qPCR data, it is expected that 90% of the *P. rapae*-induced signal on northern blots can be assigned to *VSP1* expression. First-instar (L1) *P. rapae* larvae were allowed to feed for 24 h on *Arabidopsis* plants, after which the caterpillars were removed. **Figure 1A** shows that *MYC2* transcription was induced to high levels not only in locally damaged leaves that were eaten by the caterpillars, but also in systemic leaves that were not damaged, until 6 h after removal of the caterpillars (*t* = 30 h). At later time points *MYC2* transcript levels decreased but remained elevated in comparison to non-infested control plants. The level of *P. rapae*-induced *MYC2* expression was strikingly similar in damaged and undamaged leaves. In locally damaged leaves of *P. rapae*-infested wild-type Col-0 plants, *MYC2* transcription coincided with activation of the MYC-branch marker genes *VSP1/2*, which peaked also at 6 h after removal of the caterpillars (**Figure 1B**). However, in systemic undamaged tissue *VSP1/2* transcription was remarkably lower. These results show that despite the fact that local and systemic tissues accumulated similar levels of *MYC2* transcripts, subsequent activation of the downstream target genes *VSP1/2* of the MYC-branch is severely reduced in systemic tissue.

In herbivore-damaged leaves, activation of the MYC-branch of the JA pathway results in suppression of the ERF-branch (Verhage et al., 2011). MYC2-impaired *jin1-2* and *jin1-7* mutant plants thus displayed enhanced expression of the ERF-branch regulator *ORA59* and the ERF-branch marker gene *PDF1.2* in local insect-damaged leaves (**Figures 1B, C**; Verhage et al., 2011). In systemic tissue of infested *jin1* mutant plants no elevation in ERF-branch activity was observed (**Figures 1B, C**). In fact, the levels of *PDF1.2* and *ORA59* expression were as low as in systemic tissues of Col-0 plants that were infested by *P. rapae*. Apparently, the ERF-branch of the JA pathway did not become activated in systemic leaves, neither in wild-type Col-0, nor in MYC2-impaired *jin1* mutant plants. Hence, the observed differences in JA-regulated gene expression in damaged versus undamaged leaves are confined to transcriptional activation of *MYC2*, without downstream consequences on *VSP1/2* induction and ERF-branch repression.

### DIFFERENT SIGNAL SIGNATURES IN DAMAGED VERSUS UNDAMAGED LEAVES OF *P. rapae*-INFESTED PLANTS

**FIGURE 2:**
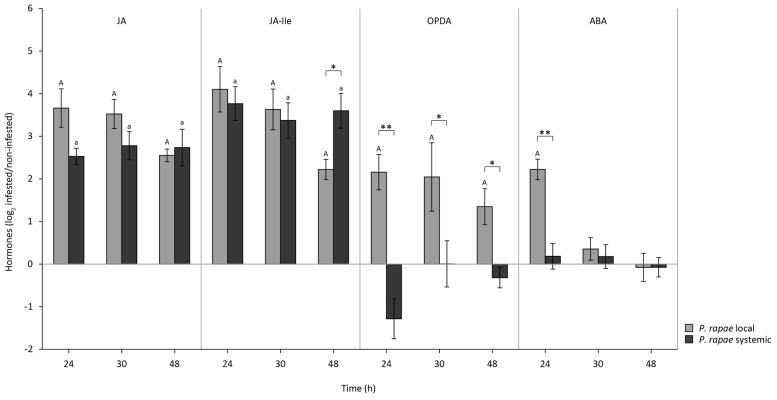
**Production of JA, JA-Ile, OPDA, and ABA in local damaged and systemic undamaged leaves of Col-0 plants upon herbivory by *P. rapae*.** Levels of JA, JA-Ile, OPDA, and ABA in leaves of Col-0 plants on which *P. rapae* larvae had been allowed to feed for 24 h. Compound levels were measured by Triple Quad LC/MS/MS. Depicted are log_2_-transformed fold induction values of the compound levels (±SD) in local damaged and systemic undamaged leaves of the same *P. rapae*-infested plants as compared to the levels in leaves of non-infested plants that were harvested at the same time points. Statistics were performed on log_2_-transformed data. Letters indicate statistically significant differences in compound levels between leaves of non-infested control plants and damaged local leaves (capital letters) and undamaged systemic leaves (small letters) of *P. rapae*-infested plants. Data were analyzed per time point using Student’s *t*-test (*P* < 0.05). Asterisks indicate statistically significant differences in compound levels between the damaged (local) and undamaged (systemic) leaves of *P. rapae*-infested plants. Data were analyzed per time point using Student’s *t*-test (**P < 0.01; *P < 0.05).

The arsenal of defense responses that is triggered by the JA pathway depends on the different isoforms of JA and on the hormonal context in which bioactive JAs are produced. To investigate whether the differences in JA-responsive gene expression in damaged and undamaged leaves of *P. rapae*-infested plants may be related to differences in the hormonal signal signature, we monitored the accumulation of JA, its precursor OPDA, the biologically highly active amino acid conjugate JA-Ile, and ABA as it is a modulator of JA signaling and can mediate resistance to generalist herbivores. Again, L1 larvae were allowed to feed for 24 h after which they were removed from the leaves. Hormone levels were measured 0, 6, and 24 h later (*t* = 24, 30, and 48). JA, JA-Ile, OPDA, and ABA levels increased significantly in locally *P. rapae*-damaged leaves (**Figure 2**). JA and JA-Ile levels also rose in systemic undamaged tissue of the same plants and reached similar levels as in herbivore-damaged leaves (**Figure 2**). In contrast, no rise in OPDA and ABA levels was detected in systemic undamaged tissue (**Figure 2**). These results demonstrate that the signature of JA, JA-Ile, OPDA, and ABA as detected in damaged leaf tissue of herbivore-infested plants differs from that in distal undamaged tissue due to a lack in increase of ABA and OPDA.

### *Pieris* rapae FEEDING INDUCES *MYC2* IN UNDAMAGED LEAVES AND PRIMES FOR ENHANCED *P. rapae*- AND ABA-INDUCED *MYC2* AND *VSP1*

**FIGURE 3:**
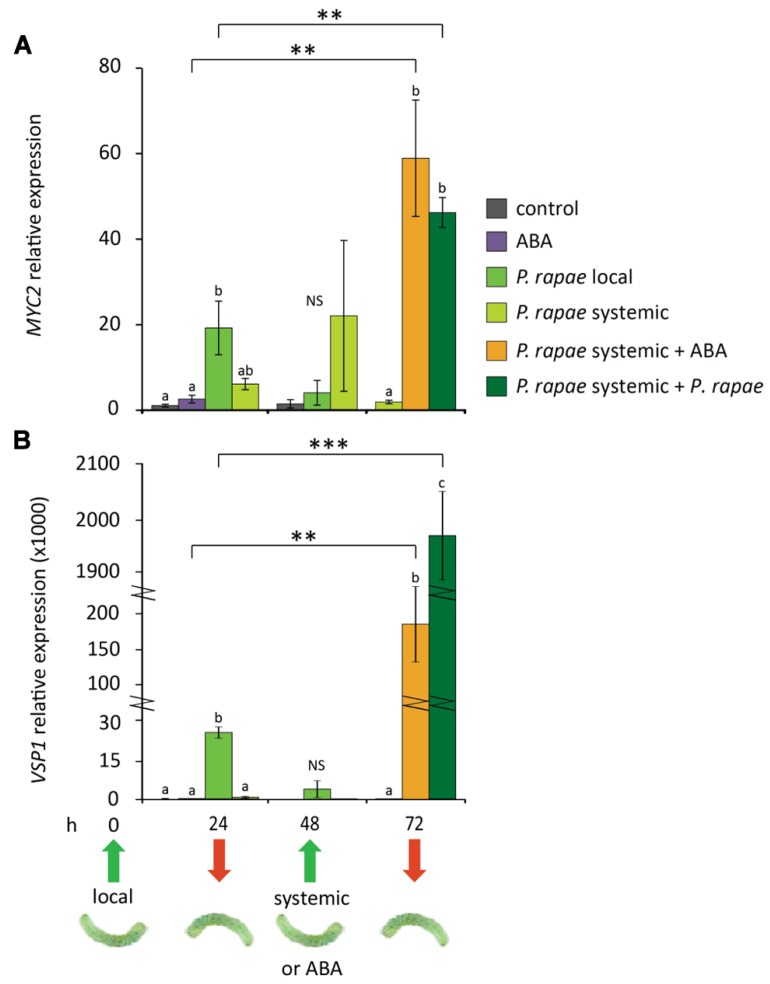
**Effects of *P.* rapae feeding and ABA treatment on expression of the MYC-branch in undamaged leaves of pre-infested Col-0 plants.** RT-qPCR analysis of **(A)** *MYC2* and **(B)** *VSP1* transcript levels (relative to non-infested control at 24 h) in *P. rapae*-infested Col-0 plants. First-instar larvae were allowed to feed for 24 h after which they were removed (*t* = 24 h). Damaged (*P. rapae* local) and undamaged (*P. rapae* systemic) leaves were harvested for analysis at different time points after removal of the *P. rapae* larvae. In addition, at *t* = 48 h, undamaged systemic leaves were challenged with fresh first-instar larvae or treated with 10 μM ABA. Treated systemic leaf tissue was harvested 24 h later (*t* = 72 h) for gene expression analysis. For comparison, non-infested plants received ABA treatment as well. Depicted are the average expression levels (±SE) of three biological replicates, consisting of five leaves each. Different letters indicate statistically significant differences between treatments at specific time points (ANOVA, Tukey *post hoc* test; *P* < 0.05; NS, not significant). Asterisks indicate statistically significant differences between (ABA or *P. rapae*)-treated leaves treatment of undamaged (*P. rapae* systemic) leaves of *P. rapae*-infested plants and the same treatment of leaves of non-preinfested plants (Student’s *t*-test; **P < 0.01; ***P < 0.001).

Previously, we demonstrated that systemic priming for enhanced JA-regulated defenses by ISR-inducing beneficial root-colonizing rhizobacteria is associated with enhanced expression of *MYC2* in above-ground plant parts, without a direct effect on the expression of downstream JA-responsive target genes (Pozo et al., 2008; Van der Ent et al., 2009a). Moreover, Abe et al. (2003) found that overexpression of *MYC2* primes the plants for enhanced sensitivity to ABA, resulting in enhanced expression of ABA-responsive genes upon exogenous ABA application. We therefore hypothesized that the observed systemic increase of *MYC2* transcripts in herbivore-damaged plants is part of a herbivore-induced priming response that may lead to an accelerated defense response after secondary herbivore attack when ABA levels rise due to damage of the tissue. To investigate this, we monitored the expression of *MYC2* and *VSP1* at different time points in damaged and undamaged tissue of *P. rapae*-infested Col-0 leaves, before and after challenge with a secondary infestation by *P. rapae* or exogenous application of 10 μM ABA. **Figure 3** shows that *MYC2* and *VSP1* genes were locally induced at 24 h after *P. rapae* larvae were placed on the leaves, which was the time point that the larvae were removed, after which transcription leveled off at 48 h. In systemic tissue, *MYC2* mRNA levels were increased sixfold at 24 h and 20-fold at 48 h, whereas expression of the MYC2-regulated gene *VSP1* was not increased systemically. These findings are in line with the results shown in **Figure 1**. At 48 h, fresh *P. rapae* caterpillars were allowed to feed from the plants and this secondary infestation of previously undamaged leaves of infested plants resulted in enhanced expression of *MYC2* (twofold) and especially *VSP1* (80-fold) compared to infestation of non-pre-infested plants. Also, when systemic leaves were challenge treated with exogenously applied ABA, the expression levels of *MYC2* and *VSP1* increased significantly (20- and 1600-fold, respectively) compared to ABA treatment of control plants, that by itself already led to 2- and 100-fold induction levels, respectively, compared to uninduced control plants. These results indicate that undamaged tissue of *P. rapae*-infested plants is primed for enhanced expression of *MYC2* and *VSP1* and that ABA plays an important role in the onset of the potentiated expression pattern upon challenge.

### *Pieris* rapae-INDUCED RESISTANCE IS BLOCKED IN *aba*2-1 AND *coi*1-1 MUTANTS

**FIGURE 4:**
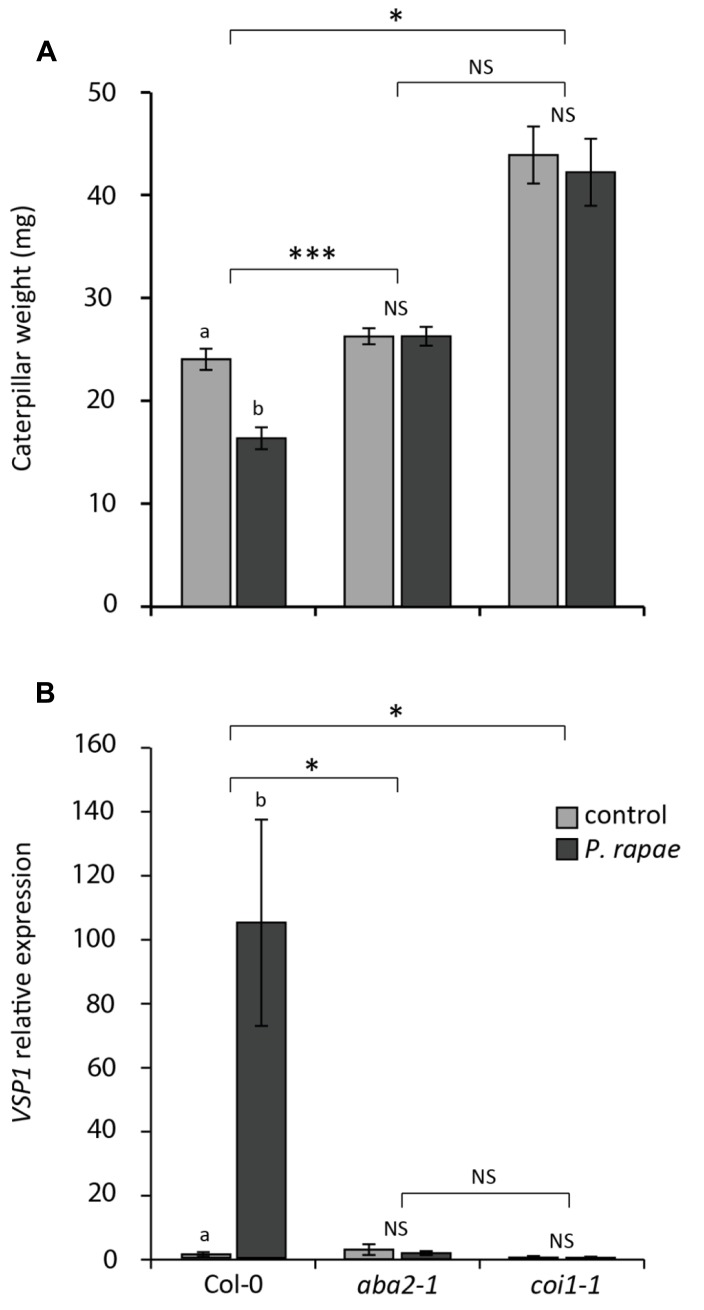
**Effect of herbivory on *P. rapae* performance on Col-0, *aba2-1*, and *coi1-1* plants. (A)** Growth of *P. rapae* larvae on herbivore-induced (*P. rapae*) and uninduced (control) Col-0, *aba2-1*, and *coi1-1* plants. A first-instar larva was allowed to feed for 24 h after which it was removed. Another 24 h later, a new first-instar larva was placed onto uninduced plants and *P. rapae*-induced plants. Caterpillar fresh weight was measured after 7 days of feeding. Feeding of a single first-instar larva for 24 h caused only minor chewing damage on usually one leaf, leaving ample tissue on all the plant genotypes for the subsequent larva to feed on for 7 days. The values presented are means ( ± SE) of 30–36 larvae that fed on similarly treated plants. Different letters indicate statistically significant differences between performance on control (non-pre-infested) and herbivore-pretreated plants per genotype (*P* < 0.05; NS, not significant); asterisks indicate statistically significant interaction between genotype × treatment (*P < 0.05; ***P < 0.001). Data were analyzed using two-way ANOVA. **(B)** RT-qPCR analysis of *VSP1* transcript levels (relative to non-infested Col-0) in non-infested (control) and *P. rapae*-infested (*P. rapae*) Col-0, *aba2-1*, and *coi1-1* plants. First-instar larvae were allowed to feed for 24 h after which they were removed and damaged leaves were harvested for analysis. Depicted are the average expression levels (±SE) of three biological replicates, consisting of five leaves each. Different letters indicate statistically significant differences between control and treatment of one genotype (*P* < 0.05; NS, not significant); asterisks indicate statistically significant interaction between genotype × treatment (*P < 0.05). Data were analyzed using two-way ANOVA.

To investigate the role of ABA in *P. rapae*-induced resistance in undamaged systemic tissue, we assessed the performance of *P. rapae* on uninduced and *P. rapae*-induced Col-0, ABA biosynthesis mutant *aba2-1* and JA unresponsive mutant *coi1-1*. As an induction treatment, an L1 larva was placed on each plant and was allowed to feed for 24 h, which resulted in minor chewing damage on usually one leaf, after which the larva was removed. Subsequently, a new L1 larva was placed on *P. rapae*-induced and on untreated control plants. After 7 days the weight of these caterpillars was determined. **Figure 4A** shows that the caterpillars weigh significantly less when fed on Col-0 plants that were pre-treated with *P. rapae* than on control Col-0 plants that were not pre-induced, confirming the findings of De Vos et al. (2006). The herbivore-induced reduction of *P. rapae* performance as observed in Col-0 plants was completely blocked in *aba2-1* and *coi1-1* mutant plants (**Figure 4A**). To investigate if the absence of this resistance effect in *aba2-1* and *coi1-1* plants coincides with reduced expression of the *VSP1* marker gene, we monitored *VSP1* transcript levels after infestation by *P. rapae*. **Figure 4B** shows that *VSP1* induction by *P. rapae* feeding is completely absent in both *aba2-1* and *coi1-1* mutant plants. Together, these results indicate that both ABA and JA play an important role in the expression of *P. rapae*-induced defenses.

## DISCUSSION

Previously, we demonstrated that herbivory by *P. rapae* on *Arabidopsis* leads to activation of the MYC-branch of the JA pathway and concomitant suppression of the ERF-branch of the JA pathway in herbivore-damaged leaves (Verhage et al., 2011). Because *P. rapae* larvae have a preference to feed from for leaf tissue that expresses the ERF-branch of the JA pathway (Verhage et al., 2011), it is thought that MYC2-mediated suppression of the ERF-branch is part of the plant’s defense strategy to limit herbivore damage. Here we investigated whether this differential JA response during herbivory is extended to systemic undamaged tissues. We demonstrated that *P. rapae* feeding induces similar levels of *MYC2* gene expression in damaged and undamaged leaves (**Figure 1**). However, in systemic undamaged leaves this did not lead to full induction of the MYC-branch of the JA-pathway, as evidenced by low *VSP1/2* induction levels and lack of MYC2-mediated suppression of the ERF-branch marker genes *ORA59* and *PDF1.2* (**Figures 1B, C**).

This observation resembles a phenomenon that is called priming for enhanced defense (Conrath et al., 2006), which is also observed during rhizobacteria-mediated ISR. Upon root colonization by beneficial ISR-inducing rhizobacteria, aboveground plant tissue acquires an enhanced level of resistance that is effective against a broad spectrum of pathogens and herbivorous insects (Van Wees et al., 2008; Pineda et al., 2010). This type of systemic induced resistance is not associated with direct activation of defense-related genes, but a large set of predominantly JA-responsive genes becomes primed for accelerated expression after pathogen or insect attack (Van Wees et al., 1999; Pozo et al., 2008; Van Oosten et al., 2008). Pozo et al. (2008) and Van der Ent et al. (2009a) demonstrated that induction of ISR elicited by *Pseudomonas fluorescens* WCS417r was associated with enhanced expression of *MYC2* in systemic leaf tissue, resembling our observation in undamaged leaves of *P. rapae*-infested plants. Moreover, MYC2-impaired *jin1* mutants were blocked in ISR and priming of JA-regulated defenses (Pozo et al., 2008), highlighting the importance of MYC2 in this type of induced resistance and in priming for enhanced defense.

Analysis of the production of JA, JA-Ile, OPDA, and ABA in local and systemic tissues revealed that the signature of these hormonal signals in undamaged leaves of *P. rapae*-infested plants is different from that observed in herbivore-damaged leaves (**Figure 2**). Levels of all four compounds rose significantly in herbivore-infested leaves, but in undamaged leaves only JA and JA-Ile levels were elevated, even to a similar extent as in damaged leaves. This correlates with the comparable levels of *MYC2* gene expression in local and systemic leaves. In contrast, neither ABA nor OPDA levels were upregulated in undamaged tissue. OPDA levels showed even a trend of decrease after 24 h of *P. rapae* feeding, which is in support of the findings of Koo et al. (2009), who showed a rapid depletion of OPDA levels in systemic tissue of *Arabidopsis* upon infliction of mechanical damage. Possibly, systemic OPDA is converted into JA and JA-Ile (Koo et al., 2009).

The lack of an increase of ABA in undamaged tissue implies that ABA is not systemically translocated from insect-infested leaves. Erb et al. (2011) found that upon belowground herbivory in maize there was a local increase in JA, OPDA, and ABA levels, comparable to our own findings on local herbivory. In systemic leaves they detected an increase in ABA levels, whereas JA and OPDA levels remained unaltered. However, this systemic increase in ABA levels is correlated to the general water stress that is inflicted by root herbivory, whereas in our setup increased levels of ABA seem strictly related to the relatively mild local wounding caused by insect feeding on generally only one leaf. Leaf wounding and herbivory are associated with leaf water loss (Aldea et al., 2005; Consales et al., 2012) and this abiotic stress may be the cause of the detected increase in ABA. In combination with other wound-induced cues the increased ABA levels may activate effective anti-herbivore defenses. In systemic tissue of mechanically damaged soybean plants there is no water loss (Aldea et al., 2005) and this may also be the case in undamaged tissue of *P. rapae*-infested *Arabidopsis*. The consequential lack of ABA increase may prevent the direct activation of costly defense responses, while instead the tissue becomes primed for the MYC-branch of JA signaling, which is a cost-efficient way of the plant to prepare itself for future attack (Van Hulten et al., 2006; Vos et al., 2013). Upon subsequent damage of the primed systemic tissue, inflicted by secondary infestation, local ABA levels rise due to water stress, which triggers full-blown anti-herbivore defenses (**Figure 3**). In this theory, water loss/ABA acts as a sensor for plants to trigger an appropriate local response or only prime systemic tissue for future induction. The difference in hormonal signal signature between local damaged and systemic undamaged tissue may be causally related to the strongly reduced activation of downstream MYC2-dependent target genes such as *VSP1/2* in the undamaged tissue.

As a regulator of the balance between the MYC- and ERF-branches of the JA defense signaling pathway (Anderson et al., 2004; Kazan and Manners, 2013) ABA impacts resistance against both pathogens and insects. Moreover, ABA plays a role in primed plant defenses against pathogens as triggered by resistance-inducing beneficial rhizobacteria and BABA (Ton et al., 2005; Van der Ent et al., 2009a). *Arabidopsis* lines that overexpress *MYC2* are hypersensitive to ABA (Abe et al., 2003). Here, we demonstrate that the *aba2-1* mutant is blocked in *VSP1* induction upon *P. rapae* feeding, demonstrating that ABA signaling is required for activation of MYC-branch-regulated responses (**Figure 4B**). Since our plants show a systemic increase in *MYC2* expression, but no increase in *VSP1/2* expression, we tested if the enhanced *MYC2* transcript levels in the systemic leaf tissue were associated with enhanced sensitivity to ABA. Therefore, we applied 10 μM ABA to undamaged leaf tissue of *P. rapae*-induced plants. Indeed, the systemically primed leaves showed enhanced expression of the MYC-branch marker genes *MYC2* and *VSP1* in response to the ABA treatment (**Figure 3**), underlining the importance of ABA in the onset of the potentiated defense response in systemically primed leaves. Taken together, these findings indicate that ABA and JA are tightly interconnected and that regulation of ABA levels in response to herbivory can modulate JA-driven defense responses (Erb et al., 2012). Besides ABA, additional signals could regulate systemic resistance induced by herbivores. Like ABA, OPDA was shown to increase only locally upon infestation and not systemically, rendering it a valid candidate for activating the primed MYC-branch in herbivory-induced systemic tissue.

Transcription factors can act as amplifiers in defense signaling cascades. Even a modest induction during priming can be sufficient to enhance the defense signaling capacity, thereby giving the primed plant tissue a “head start” during the early stages of pathogen or insect attack. To test if the undamaged tissue of *P. rapae*-infested plants is primed for future caterpillar attack, the effect of a succeeding infestation by *P. rapae* of these tissues was tested. *MYC2* gene expression was found to be induced at twofold higher levels after the second attack than at a first attack (**Figure 3A**). The expression levels of *VSP1* in *P. rapae*-challenged systemic leaves were a steep 80-fold higher compared to those observed in non-pre-infested *P. rapae*-damaged leaves (local). These results suggest that systemic undamaged tissues of *P. rapae*-infested plants are indeed primed for enhanced defense against future caterpillar attack.

Insect performance assays demonstrated that primary infestation by one *P. rapae* larva for 24 h, which resulted in only minor chewing damage on usually one leaf, was sufficient to lead to reduced growth of a secondary infesting larva on Col-0 plants, that was placed on the plant 1 day later and of whom the weight was determined 7 days later (**Figure 4A**). This result correlates with the *P. rapae*-induced priming of undamaged tissue, leading to enhanced activation of the MYC-branch upon challenge with *P. rapae*. This protective effect was completely blocked in the ABA biosynthesis mutant *aba2-1* and the JA response mutant *coi1-1*, that are both affected in MYC signaling (**Figure 4B**), indicating that functional JA and ABA pathways are both necessary for the onset of herbivore-induced resistance in undamaged systemic tissues. One could debate whether we can speak in this situation about induction of systemic resistance for two reasons. Firstly, during the pre-infestation the larva might have crawled over more than one or two leaves and thereby caused invisible additional damage to the seemingly undamaged leaves. This cannot be excluded, but it is unlikely because *P. rapae* L1 larvae usually stay on the leaf that they are placed on for the first day. Secondly, the challenge larva that was allowed to feed for 7 days did not only feed from undamaged tissue but also from the pre-damaged tissue. The amount of pre-damaged tissue was, however, estimated to be less than 5% of the total amount of tissue, so this can unlikely explain the difference in larvae performance on pre-infested versus uninduced Col-0 plants. It is a fact that during the 7 days of the performance experiment there is a continuous induction of resistance and still, one could detect a difference in insect performance due to pre-infestation 8–9 days earlier, which indicates that the herbivore-induced effect on resistance that we detected is robust.

On the *coi1-1* mutant plants the larvae grew larger than on Col-0 and *aba2-1*, confirming previous findings that JA signaling is indispensable for insect resistance (Bodenhausen and Reymond, 2007; Fernández-Calvo et al., 2011; Verhage et al., 2011). The *aba2-1* mutant did not allow enhanced growth of the *P. rapae* larvae compared to Col-0, whereas larvae of the generalist herbivore *Spodoptera littoralis* grew significantly larger on *aba2-1* (Bodenhausen and Reymond, 2007). This suggests that ABA signaling is a critical component of the resistance response against generalists, but we show that ABA also functions as an activator of primed defense responses against specialists (and possibly also generalists).

**FIGURE 5:**
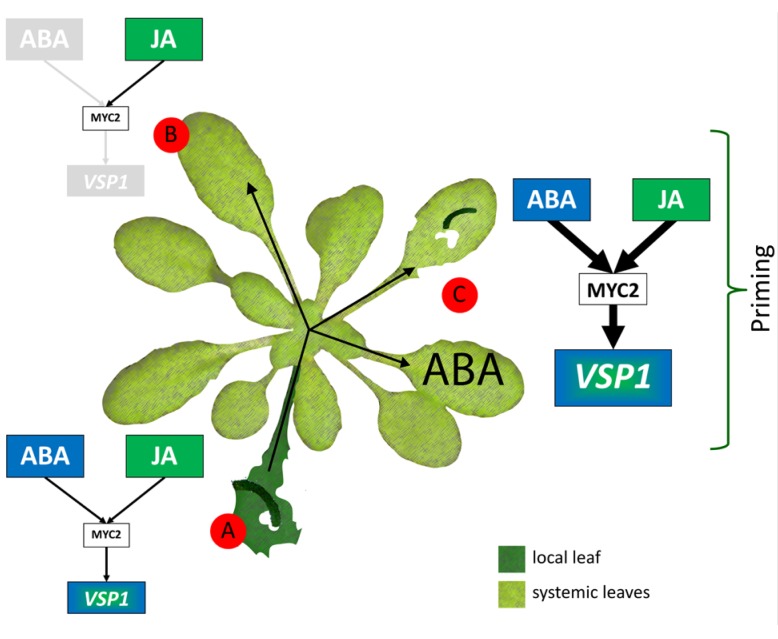
**Model of differential JA responses induced in local damaged and systemic undamaged leaves upon *P. rapae* feeding. (A)** Upon *P. rapae* feeding, local damaged leaves show upregulation of JA/JA-Ile and ABA levels and of transcriptional activity of the MYC-branch regulator gene *MYC2* and its downstream response gene *VSP1*. **(B)** In systemic undamaged leaves of *P. rapae*-infested plants, the level of JA/JA-Ile and *MYC2* gene expression is upregulated. However, the level of ABA and MYC-regulated *VSP1* transcription is not increased. **(C)** Challenge of MYC-primed systemic undamaged leaves with either *P. rapae* larvae or exogenously applied ABA leads to potentiated transcript levels of *MYC2* and particularly *VSP1*, which are much higher than those observed in local infested leaves. Furthermore, *P. rapae* performance is reduced if plants were previously infested. This indicates that undamaged tissue of *P. rapae*-infested plants is primed for enhanced defense against subsequent *P. rapae* attack and that ABA production induced upon subsequent infestation is important for the onset of potentiated expression of the MYC-branch of JA signaling.

Priming has been demonstrated to entail limited fitness costs, especially in comparison to the higher costs associated with direct activation of defenses. Moreover, the fitness costs of priming were shown to be outweighed by the enhanced resistance benefits under pathogen pressure, which suggests that priming functions as an ecological adaptation of the plant to respond faster to a hostile environment (Van Hulten et al., 2006; Walters et al., 2008; Vos et al., 2013). The data presented here point to a model (**Figure 5**) in which herbivory leads to priming of the MYC-branch of the JA pathway in systemic undamaged leaves, without fully activating costly JA-dependent defenses, for which also a local increase of ABA is required, which is likely induced by local damage-induced leaf water stress. The primed state leads to elevated activation of MYC2-dependent defenses when undamaged systemic tissue is attacked by insect herbivores. ABA is identified as a regulator of herbivore-induced resistance by activating the potentiated expression of defense responses in previously undamaged tissue upon secondary herbivore attack.

## MATERIALS AND METHODS

### PLANT GROWTH CONDITIONS

Seeds of *A. thaliana* wild-type Col-0 and mutants *jin1-2*, *jin1-7*, *aba2-1*, and *coi1-1* (Koornneef et al., 1982; Feys et al., 1994; Lorenzo et al., 2004) were sown on quartz sand and, after 2 weeks of growth, seedlings were transplanted into 60-ml pots containing a sand–potting soil mixture (5:12 v/v) that had been autoclaved twice for 20 min with a 24-h interval. Plants were cultivated in a growth chamber with an 8-h day (200 μE m^-^^2^ s^-^^1^ at 24°C) and 16-h night (20°C) cycle at 70% relative humidity. Plants were watered every other day and received half-strength Hoagland solution (Hoagland and Arnon, 1938) containing 10 μM sequestreen (CIBA-Geigy, Basel, Switzerland) once a week.

#### *Pieris* rapae ASSAYS

*Pieris rapae* (small cabbage white butterfly) was reared on white cabbage plants (*Brassica oleracea*) as described in Van Wees et al. (2013). In all experiments, L1 larvae were used. For gene expression analyses, two larvae were placed separately on fully expanded leaves of 5-week-old *Arabidopsis* plants using a fine paintbrush. The larvae were removed 24 h later and leaves were harvested at different time points after introduction of the caterpillars. Leaves damaged by caterpillar feeding (local) were harvested separately from undamaged leaves (systemic) of infested and uninfested (control) plants. Undamaged systemic leaves that received a second treatment (*P. rapae* or ABA) were distinguished from locally damaged leaves by marking the local leaves before the second treatment. Even though *P. rapae* L1 larvae commonly stayed on the one leaf on which they were introduced, they incidentally crawled to other leaves. On leaves where they would not leave visible feeding damage potential induction of additional unknown defenses cannot be excluded. Two fresh L1 larvae were placed on fully expanded, undamaged leaves of *P. rapae*-infested plants at *t* = 48 h. At *t* = 72 h the larvae were removed and systemic damaged leaves were harvested for gene expression analysis.

For the *P. rapae* performance assay, 5-week-old plants were placed in Magenta GA-7 containers with a modified mesh lid. Plants were challenged with *P. rapae* by placing one fresh L1 larva on each plant, which was then allowed to feed for 7 days. The weight of each individual larva was measured using a microbalance. To determine herbivore-ISR, each plant was exposed to herbivory by a single L1 larva for 24 h after which the larva was removed. At *t* = 48 h, plants were challenged with one fresh L1 larva, which was allowed to feed on the plants for 7 days, as described above. In all experiments there was ample tissue for the caterpillars to feed from for 7 days.

### ABA TREATMENT

Undamaged control plants and *P. rapae*-infested plants were treated with ABA (Sigma, Steinheim, Germany) by dipping the plants in a solution containing 10 μM ABA and 0.015% (v/v) Silwet L77 (Van Meeuwen Chemicals BV, Weesp, Netherlands). ABA was added to the solution from a 1000-fold concentrated stock in 96% ethanol.

### RNA EXTRACTION AND NORTHERN BLOT ANALYSIS

Total RNA was isolated as described by Van Wees et al. (1999). For northern blot analyses, 10 μg of RNA was denatured using glyoxal and dimethyl sulfoxide (Sambrook et al., 1989), electrophoretically separated on a 1.5% agarose gel, and blotted onto Hybond-N^+^ membranes (Amersham, ‘s-Hertogenbosch, Netherlands) by capillary transfer. The electrophoresis and blotting buffer consisted of 10 and 25 mM sodium phosphate (pH 7.0), respectively. To check for equal loading, rRNA bands were stained with ethidium bromide. Northern blots were hybridized with gene-specific probes for *PDF1.2*, *VSP1/2*, and *MYC2* as described (Leon-Reyes et al., 2010; Verhage et al., 2011). After hybridization with α-[^32^P]-dCTP-labeled probes, blots were exposed for autoradiography and signals were quantified using a Bio-Rad Molecular Imager FX with Quantity One software (Bio-Rad, Veenendaal, Netherlands). The AGI numbers for the genes studied are indicated in the primer table below. All gene expression analyses have been repeated with similar results.

### RT-qPCR

SuperScript^TM^ III Reverse Transcriptase was used to convert DNA-free total RNA into cDNA. PCR reactions were performed in optical 96- or 384-well plates (Applied Biosystems) with an ABI PRISM^™^ 7900 HT sequence detection system, using SYBR^™^ Green to monitor to synthesis of double-stranded DNA. A standard thermal profile was used: 50°C for 2 min, 95°C for 10 min, 40 cycles of 95°C for 15 s and 60°C for 1 min. Amplicon dissociation curves were recorded after cycle 40 by heating from 60 to 95°C with a ramp speed of 1.0°C min^-^^1^. Transcript levels were calculated relative to the reference gene At1g13320 (Czechowski et al., 2005) using the 2^-^^Δ^^Δ^^Ct^ method described previously (Livak and Schmittgen, 2001; Schmittgen and Livak, 2008).

The following primers were used:

**Table:** 

At1g13320	Forward: 5′-TAA CGT GGC CAA AAT GAT GC-3′
	Reverse: 5′-GTT CTC CAC AAC CGC TTG GT-3′
*ORA59* (At1g06160)	Forward: 5′-TTCCC CGAG AACTC TTCTT-3′
	Reverse: 5′-GCCTG ATCAT AAGCG AGAGC-3′
*PDF1.2 (At5g44420)*	Forward: 5′-TTTGC TGCT TTCGA CGCAC-3′
	Reverse: 5′-CGCAA ACCC CTGAC CATG-3′
*MYC2 (At1g32640)*	Forward: 5′-GATGA GGAG GTGAC GGATA CGGAA-3′
	Reverse: 5′-CGCT TTACC AGCTA ATCC CGCA-3′
*VSP1* (At5g24780)	Forward: 5′-TCGA AGTT GACG CAAG TGGT-3′
	Reverse: 5′-GGGG ACAA TGCC ATGA AGAT-3′

### HORMONE ANALYSIS

For JA, JA-Ile, OPDA, and ABA concentration analysis, 0.5 g of leaf tissue was ground in a mortar with liquid nitrogen. The samples of *P. rapae*-damaged leaves (local) and -undamaged leaves (systemic) originated from the same plants. The extraction and hormone analysis was performed as described (López-Ráez et al., 2010); 2 ml of cold ethyl acetate containing [2H6]-ABA was added to the samples at the start of the extraction as an internal standard (0.25 nmol) in order to calculate the recovery of the hormones measured. Hormone levels were analyzed by LC–MS on a Varian 320 Triple Quad LC/MS/MS. Ten microliters of each sample was injected onto a Pursuit column (C18; 5 μm, 50 × 2.0 mm; Varian) that was connected to a precolumn (Pursuit Metaguard C18; 5 μm; 2.0 mm). Multiple reaction monitoring was performed for parent-ions and selected daughter-ions after negative ionization: JA 209/59 (fragmented under 12V collision energy), JA-Ile 322/130 (fragmented under 19V collision energy), OPDA 291/165 (fragmented under 18V collision energy), and ABA 263/153, ABA-D_6_ 269/159 (both isoforms of ABA fragmented under 9V collision energy). The mobile phase comprised solvent A (0.05% formic acid) and solvent B (0.05% formic acid in MeOH) with settings as described (Diezel et al., 2009). The retention time of each compound was confirmed with pure compounds (ChemIm Ltd, Olomouc, Czech Republic). The surface area for each daughter-ion peak was recorded for the detected analytes. The analytes were quantified using standard curves made for each individual compound.

## Conflict of Interest Statement

The authors declare that the research was conducted in the absence of any commercial or financial relationships that could be construed as a potential conflict of interest.
